# A Blood and Biochemical Indicator-Based Prognostic Model Predicting Latent Tuberculosis Infection: A Retrospective Study

**DOI:** 10.3390/tropicalmed10060154

**Published:** 2025-06-01

**Authors:** Beibei Qiu, Zhengyuan Xu, Yanqiu Huang, Ruifen Miao

**Affiliations:** 1 Department of Chronic Communicable Disease, Nanjing Municipal Center for Disease Control and Prevention, Nanjing 210009, China; 18351995030@163.com (B.Q.); zhengyuanxu00@163.com (Z.X.); 2 School of Public Health, Shanghai Jiao Tong University School of Medicine, Shanghai 200025, China; huangyanqiu@sjtu.edu.cn

**Keywords:** latent tuberculosis infection, immune cell, clinical indicators, eosinophils, uric acid

## Abstract

Background and Objectives: Abnormal blood and biochemical indicators could increase the risk of infectious diseases. However, the association between blood together with biochemical indicators and latent tuberculosis infection (LTBI) has not been well confirmed. Materials and Methods: Our aim was to assess the role of blood and biochemical indicators in the risk of LTBI. We enrolled 965 freshmen who were originating from tuberculosis key areas of a college in Nanjing. We used logistic regression models, restricted cubic spline (RCS), and nomograms to evaluate the association between blood and biochemical indicators and LTBI. In addition, calibration curves were performed to evaluate the quality of the model. Results: Among these 965 participants, 311 were diagnosed as LTBI according to TST. Multivariate models showed that the population with an eosinophils percentage around <0.5% (OR: 2.82, 95% CI: 1.39–5.74, *p* = 0.004) and 0.5–5% (OR: 2.78, 95% CI: 1.07–7.23, *p* = 0.036) were positively associated with LTBI. Elevated uric acid levels (OR: 1.01, 95% CI: 1.00–1.02, *p* = 0.047) were significantly associated with LTBI. In addition, participants with a history of tuberculosis exposure (OR: 3.26, 95% CI: 1.39–7.66) and a history of tuberculosis (OR: 10.92, 95% CI: 1.24–96.08) were also positively correlated with LTBI. Conclusions: Eosinophils percentage and uric acid are associated with LTBIs. Participants who have tuberculosis exposure history and tuberculosis history are the critical target population.

## 1. Introduction

Tuberculosis is a chronic infectious disease caused by *Mycobacterium tuberculosis* (*M. tb*), which is characterized by tuberculous lesions of the respiratory system. According to the World Health Organization (WHO)’s latest report [1], the global incidence rate of tuberculosis in 2023 reached 134/100,000, with 1.25 million deaths and 662,000 infected with HIV. The number of new tuberculosis cases in China in 2024 was 702,500, and the estimated incidence rate of tuberculosis was 49.88/100,000. China ranked third among the 30 countries with high burden of tuberculosis around the world, next only to India and Indonesia.

Latent tuberculosis infection (LTBI) is an infection of *Mycobacterium tuberculosis* in the body without evidence of active tuberculosis in clinical symptoms, bacteriology, or imaging. It is estimated that 1/4 of the global population is infected with tuberculosis. Moreover, the latent infected population is a huge potential “patient repository”, of which about 5–10% will progress to active tuberculosis in their lifetime [2,3]. Approximately 350 million people in China are infected with LTBI, making it one of the countries with the heaviest burden of LTBI globally [4].

The impaired immune response of people with LTBI would increase their risk of progressing into active tuberculosis. Even more significantly, macrophage activation, CD4^+^T cells, CD8^+^T cells, IFN-γ, and TNF-α may also promote the pathogenic risk of latent infections [5]. A study showed that LTBI substantially changed the immune cell compartments in the BALF, especially for the three subsets of macrophages, monocyte macrophage (MM)-CCL23, MM-FCN1, and MM-SPP1 [6], which were found to be associated with the disease status of tuberculosis infection. An American study indicated that LTBI individuals uniquely display ongoing immune activation and robust CD4^+^T cell recall responses in blood and the lungs [7]. However, there were no studies on the association between blood immune cell markers and the risk of LTBI.

The reported incidence of tuberculosis in Chinese students is about one-third of the whole population, and the 15–24 year old age group accounts for about 85% of the total cases reported by students. This means the number of cases in high school and college students is significant, with the age group including 18-year-olds accounting for the highest proportion [8]. School is a place where students are highly concentrated. As a common respiratory infectious disease, a tuberculosis epidemic in schools not only causes serious damage to students’ physical and mental health, but also has an important impact on school teaching order and environmental stability [9]. Moreover, outbreaks of MDR-TB have been reported frequently in school tuberculosis outbreaks, which makes tuberculosis control in schools more difficult [10,11]. Tuberculosis screening is an important part of the school tuberculosis prevention and control strategy [12,13]. Nanjing is located in East China; the number of universities ranks third in the country, the population mobility is large, and clusters of tuberculosis in schools occur occasionally.

The purpose of this study was to investigate the epidemiological association between blood, together with biochemical indicators, and LTBI based on tuberculosis screening of college freshmen in Nanjing. It could provide a new idea for screening LTBI and preventing tuberculosis.

## 2. Materials and Methods

### 2.1. Study Population and Data Source

This study was carried out in a university in Nanjing in September 2023. Participants were all new students from key tuberculosis areas. The inclusion criteria were given as follows: (1) freshmen from the key area of tuberculosis; (2) signed an informed consent; (3) participated in the tuberculin skin test and clinical biochemical examination; and (4) completed the questionnaire. This study was conducted in accordance with the Declaration of Helsinki.

Participants included in our study were investigated using questionnaires to collect demographic and sociological information. Tuberculin skin tests and clinical biochemical examinations were performed immediately.

### 2.2. Tuberculin Skin Test (TST)

Intradermally, 0.1 mL of PPD preparation was injected at l/3 above the curved side of the left forearm, and the results were examined within 48 to 72 h. Result determination included the following: (1) negative: average diameter of the induration < 5 mm or without response; (2) generally positive: average diameter between 5 mm–10 mm; (3) moderately positive: average diameter between 10–15 mm; and (4) strongly positive: ≥15 mm or <15 mm but with double rings, blisters, necrosis and lymphangitis. According to national guidelines (WS288-2017), the PPD reaction with a hardened diameter ≥ 10 mm was defined as LTBI.

### 2.3. Clinical Biochemical Examination

The clinical examination items include blood routine tests and liver and kidney function tests. We selected neutrophil percentage, eosinophils percentage, basophils percentage, absolute value of lymphocytes, absolute value of monocytes, lymphocytes percentage, monocytes percentage, uric acid, blood urea nitrogen, alanine aminotransferase and creatinine as indicators to explore the association with the risk of LTBI.

### 2.4. Statistical Analysis

Logistic regression models were used to estimate factors associated with LTBI. We included the factors with a *p*-value < 0.20 in the univariate logistic regression model in the multivariate analysis, and forcibly included gender to reduce the influence of gender on these factors. The odds ratio (OR) and corresponding 95% confidence interval were calculated. The above analyses were conducted using R 4.4.2 (accessed from February to May 2025 (https://www.r-project.org)). *p*-values < 0.05 were considered statistically significant in our analyses. RCS was used to visualize the association between immune cell indicators and LTBI. In addition, we also adopted a calibration curve to determine the quality of the model. A nomogram was used to evaluate the possibility of the demographic characteristics-adjusted multivariable model predicting the risk of LTBI.

### 2.5. Ethics Considerations

The studies involving human participants were reviewed and approved by the Ethics Committee of Nanjing Municipal Center for Disease Control and Prevention. Written informed consent to participate in this study was provided by the participants. Data analyses were done anonymously, using unique study numbers.

## 3. Results

### 3.1. Demographic Characteristics of Participants

**Figure 1:**
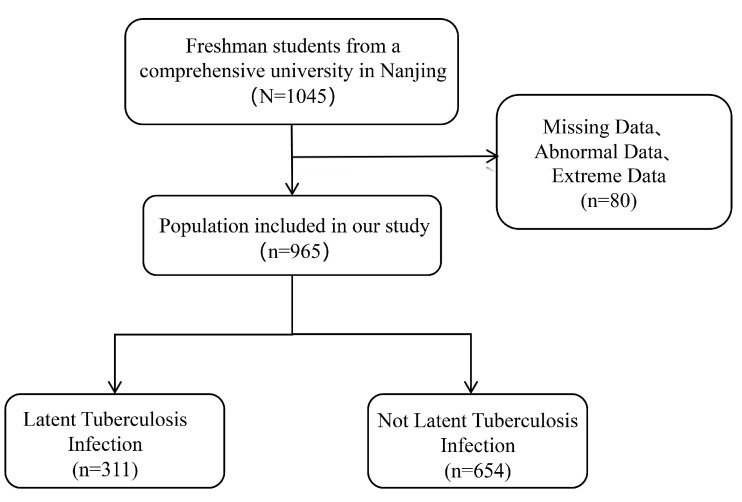
Flowchart of participants.

Aggregately, 1045 freshmen who were derived from tuberculosis key areas were enrolled in our study. These participants accomplished questionnaire surveys, tuberculin skin tests, and biochemical index examinations. After excluding missing data, abnormal data, and extreme data, 965 individuals were ultimately included in the analysis. According to the tuberculin skin test, 311 participants (32.23%) were identified as having tuberculosis latent infections, while another 654 (67.77%) were classified as not having tuberculosis latent infections (Figure 1).

### 3.2. Demographic Characteristics and Clinical Information

In our study, 467 males (48.39%) and 498 females (51.61%) were included. Of these, 24 participants (2.49%) had a history of exposure to tuberculosis. The number of Han ethnicity participants was 701 (72.64%). Furthermore, there were 143 people (14.82%) who had a hospitalization history. In our study population, 7 participants (0.73%) had a history of tuberculosis. The number of participants with a clear history of BCG vaccination was 886 (91.81%) (Table 1).

Neutrophil percentage, eosinophils percentage, basophils percentage, absolute value of lymphocytes, absolute value of monocytes, lymphocytes percentage, and monocytes percentage were selected as indicators of immune cell function. Uric acid, Blood urea nitrogen, alanine aminotransferase, and creatinine were selected as indicators of liver and kidney function.

The distribution of the central and discrete trends of these data is shown in Table 2.

### 3.3. Factors Related to LTBI

Firstly, we attempted to incorporate clinical indicators and demographic characteristics that could potentially be related to LTBI into a univariate logistic regression analysis. The results showed that the association with LTBI was higher in participants with an eosinophils percentage of <0.5% compared to those with >5% (OR: 2.65, 95% CI: 1.33–5.29); a significant association with LTBI was also observed in the population with an eosinophils percentage between 0.5–5% (OR: 2.75, 95% CI: 1.08–7.00). Additionally, participants with a history of tuberculosis were more likely to have a positive skin test result compared to those without a history of tuberculosis (OR: 12.85, 95% CI: 1.54–107.17). A history of tuberculosis exposure also had a positive association with LTBI (OR: 3.63, 95% CI: 1.57–8.39). We included the factors with a *p*-value < 0.20 in the univariate logistic regression model in the multivariate analysis, and forcibly included gender to reduce the influence of gender on these factors. The results showed that the population with an eosinophils percentage <0.5% (OR: 2.82, 95% CI: 1.39–5.74) and 0.5–5%(OR: 2.78, 95% CI: 1.07–7.23) had a positive association with LTBI. Increased uric acid levels (OR: 1.01, 95% CI: 1.00–1.02) were significantly associated with LTBI. In addition, the population with a history of tuberculosis exposure (OR: 3.26, 95% CI: 1.39–7.66) was also positively associated with LTBI. Participants with a history of tuberculosis (OR: 10.92, 95% CI: 1.24–96.08) were positively associated with LTBI (Table 3).

**Figure 2:**
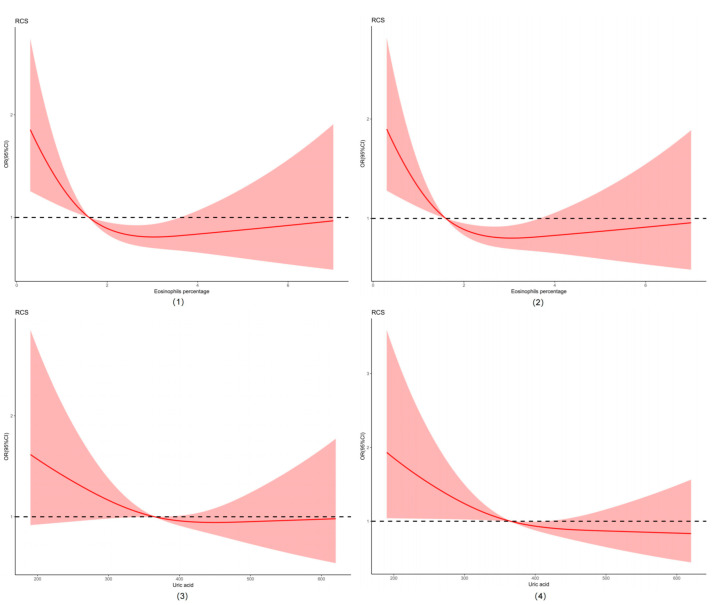
Restricted cubic spline (RCS) of eosinophils percentage and uric acid. Abbreviations: (**1**) restricted cubic spline of eosinophils percentage; (**2**) restricted cubic spline of eosinophils percentage by gender stratification; (**3**) restricted cubic spline of uric acid; (**4**) restricted cubic spline of uric acid by gender stratification.

We plotted the restricted cubic spline (RCS) curve using eosinophils percentage and uric acid due to their significant differences in the analysis results. Eosinophils percentage and uric acid levels of the participants by gender stratification, which are also shown in Figure 2.

### 3.4. Prediction of the Risk of LTBI

**Figure 3:**
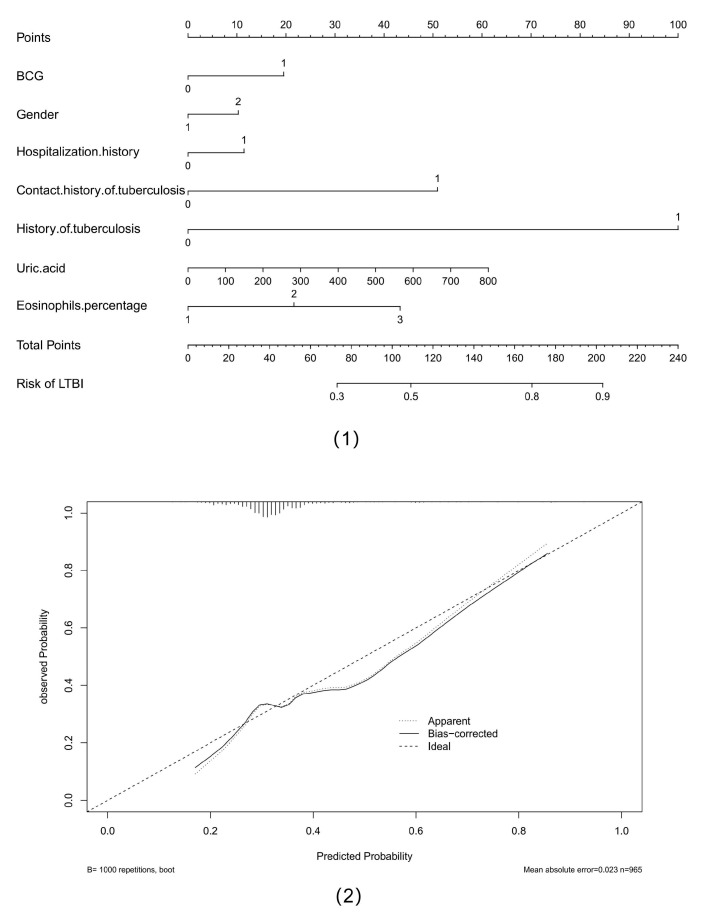
Nomogram of predicting the risk of latent infection of tuberculosis and calibration curve of multiple models. Abbreviations: (**1**) nomogram of predicting the risk of latent infection of tuberculosis; (**2**) calibration curve of multiple models.

Furthermore, our study attempted to use the nomogram to clinically predict the risk of LTBI using associated clinical indicators and demographic characteristics. A population nomogram prediction model was constructed based on seven variables, including BCG, gender, hospitalization history, history of tuberculosis, contact history of tuberculosis, eosinophils percentage, and uric acid, according to multivariate logistic regression analysis. Each variable was assigned a score ranging from 0 to 100. The scores of each variable were summarized. The total score was determined based on the individual scores calculated using the nomogram; most participants in the present study had a risk of LTBI that ranged from 30% to 90%. We also used a calibration curve to reflect the quality of our model’s predictions. The mean absolute error was 0.023, which was relatively low. The results are shown in Figure 3.

## 4. Discussion

This study attempts to explore the intrinsic association between clinical immunity with biochemical indicators and LTBIs among college students. The demographic characteristics-adjusted multivariable model detected that the population with an eosinophils percentage between <0.5% (*p* = 0.004) and 0.5–5% (*p* = 0.036) was positively associated with LTBI. Elevated uric acid levels (*p* = 0.047) were significantly associated with LTBI. In addition, participants with a history of tuberculosis exposure and a history of tuberculosis were also positively correlated with LTBI. We believed that these findings would provide a possible theoretical basis for screening LTBI.

In recent years, identifying LTBI and preventing it from developing into tuberculosis is the cornerstone of tuberculosis control and is also one of the important contents to accomplish the purpose of “End tuberculosis” in 2035 [14,15,16]. A longitudinal cohort study spanning 10 years pointed out that people had the highest risk of progressing to tuberculosis within 2 years of infection with *Mycobacterium tuberculosis* (1.5% annual risk) [17]. LTBI is not a stable state; intermittent, transient, or progressive episodes of mycobacterial replication can lead to incipient, then subclinical, and finally active TB disease [18]. Screening for LTBI in high-risk populations is an extremely important strategy for the prevention and control of active tuberculosis [19]. LTBI is a huge reservoir for active tuberculosis cases [2,20]. In fact, since most new tuberculosis cases are caused by remote reactivation of LTBI rather than recent infection, strengthening LTBI screening and treatment strategies is considered to be a key component of eliminating tuberculosis, especially in an environment with low tuberculosis prevalence.

A longitudinal study of immune responses in healthy *Mycobacterium tuberculosis*-infected people could increase a deeper understanding of the foundation of protective immunity. The change of blood-based biomarkers could capture the process by which *Mycobacterium tuberculosis* infects the human immune system. Some studies previously identified differences in *Mycobacterium tuberculosis*-specific T-cell populations and cytokine secretion profiles in patients with tuberculosis versus those with LTBI [21]. A study that explored blood cells and interferon-gamma level correlation in LTBI recruited 88 household contacts of tuberculosis pulmonary patients and compared blood cells. They found that there were no significant changes in blood cells between infected and noninfected individuals [22]. Multiple studies have shown that neutrophils may be associated with alternative antigen presentation for T-cells, the release of chemokines, and the induction of granuloma formation after the invasion of *Mycobacterium tuberculosis* into the body [22,23]. Eosinophils showed bactericidal potential through phagocytosis, respiratory burst, and mobilization of cytotoxic proteins in the presence of bacterial infection [24,25]. It indicated that the cells have a protective effect against bacterial infection. Another study found that eosinophils are among the earliest cells to sense and respond to *Mtb* infection in alveolar macrophages from the circulation. Moreover, cell-intrinsic expression of the oxysterol receptor GPR183 plays a role in the migration of eosinophils to lung tissue [26]. A study found that 5′-NT, uric acid, globulin, creatinine, cystatin C, and AST were key predictors for evaluating the therapeutic effect of tuberculosis [27]. It is worth noting that uric acid reflects the level of renal function. It is possible that renal function status may play a promoting role in the development process of tuberculosis. Uric acid has great clinical significance in the treatment and prognosis of tuberculosis [28]. However, there were almost no studies on the association between uric acid and LTBI. Our study results filled this gap. We also found that a tuberculosis exposure history was a high risk for LTBI. Therefore, it is urgent and indispensable to strengthen screening for latent infection among close contacts of tuberculosis. This finding is consistent with numerous authoritative studies [29,30].

However, there was still no systematic and comprehensive study exploring the relation between multiple categories of blood together with biochemical indicators and LTBI currently. This study explored the possibility of blood and biochemical indicators as biomarkers for the diagnosis of LTBI and their associations. Our study has the following innovations: firstly, participants of the comprehensive college come from central and western China, with good representativeness. The proportion of a non-Han population was 27.36%. Secondly, our study attempts to analyze the potential of blood and biochemical indicators as biomarkers and their association with LTBI. The study results can provide certain theoretical values for future risk prediction and diagnosis of LTBI. Thirdly, to a certain extent, our results could reflect the epidemiological characteristics of LTBI in China and effectively carry out scientific prevention and control of tuberculosis. While some shortcomings still exist in this study. The tuberculin skin test, as a classic method for judging LTBI, would result in a certain degree of bias due to the operator and observer. It may result in errors in the study results.

## 5. Conclusions

Our results found that eosinophils percentage and uric acid have association with LTBI. Participants who have a tuberculosis exposure history and a tuberculosis history are the critical target population.

## Figures and Tables

**Table 1:** Demographic characteristics of participants.

Variables		n	%
Gender	Male	467	48.39
	Female	498	51.61
Contact history of tuberculosis	Yes	24	2.49
	No	941	97.51
Hospitalization history	Yes	143	14.82
	No	822	85.18
Ethnicity	Han	701	72.64
	Non-Han	264	27.36
History of tuberculosis	Yes	7	0.73
	No	958	99.27
BCG	Yes	886	91.81
	Self-statement: None	79	8.19

**Table 2:** Clinical information of participants.

Variables	Minimum Value	Q25	Median Value	Average Value	Q75	Maximum Value
Neutrophil percentage	33.70	54.75	60.10	60.08	65.20	81.30
Eosinophils percentage	0.10	1.00	1.60	1.94	2.50	9.90
Basophils percentage	0.00	0.30	0.40	0.49	0.60	6.40
Absolute value of lymphocytes	0.62	1.67	2.00	2.04	2.37	4.31
Absolute value of monocytes	0.02	0.32	0.39	0.41	0.48	1.21
Lymphocytes percentage	11.30	26.40	31.10	31.28	36.00	52.80
Monocytes percentage	2.40	5.15	6.00	6.17	7.00	9.99
Uric acid	31.00	309.00	365.00	374.91	432.00	788.00
Blood urea nitrogen	1.36	3.55	4.25	4.36	5.05	9.39
Alanine aminotransferase	2.50	9.60	13.30	19.26	21.25	99.99
Creatinine	39.00	58.00	68.00	68.99	79.00	116.00

**Table 3:** Demographic characteristics-adjusted and multivariable analysis in participants assessing characteristics associated with latent tuberculosis infection.

Variables		Univariate Analysis		Multivariate Analysis	
		cOR (95% CI)	*p*-Value	aOR (95% CI)	*p*-Value
Age		0.99 (0.81–1.20)	0.906		
Gender	Male	0.97 (0.74–1.27)	0.836	0.78 (0.55–1.10)	0.150
	Female	reference		reference	
Ethnicity	Han	1.22 (0.89–1.66)	0.212		
	Non-Han	reference			
Neutrophil percentage		1.01 (0.99–1.03)	0.376		
Eosinophils percentage	<0.5%	2.65 (1.33–5.29)	0.006	2.82 (1.39–5.74)	0.004 *
	0.5–5%	2.75 (1.08–7.00)	0.034	2.78 (1.07–7.23)	0.036 *
	>5%	reference		reference	
Basophils percentage	0~1%	1.32 (0.72–2.42)	0.371		
	>1%	reference			
Absolute value of lymphocytes		0.86 (0.66–1.11)	0.245		
Absolute value of monocytes		0.84 (0.30–2.35)	0.736		
Lymphocytes percentage		0.99 (0.97–1.01)	0.201		
Monocytes percentage		0.98 (0.89–1.08)	0.654		
Uric Acid		1.01 (1.00–1.02)	0.130	1.01 (1.00–1.02)	0.047 *
Blood Urea Nitrogen		1.03 (0.92–1.16)	0.578		
Alanine aminotransferase		1.00 (0.99–1.01)	0.984		
Creatinine		1.00 (0.99–1.01)	0.699		
BCG	Yes	1.55 (0.91–2.65)	0.107	1.62 (0.94–2.78)	0.084
	Self-statement: None	reference		reference	
History of tuberculosis	Yes	12.85 (1.54–107.17)	0.018	10.92(1.24–96.08)	0.031 *
	No	reference		reference	
Contact history of tuberculosis	Yes	3.63 (1.57–8.39)	0.003	3.26 (1.39–7.66)	0.007 *
	No	reference		reference	
Hospitalization history	Yes	1.38 (0.96–2.00)	0.085	1.34 (0.91–1.96)	0.138
	No	reference		reference	

Abbreviations: OR, odds ratio; CI, confidence interval. * indicates the *p*-value of the multivariate analysis was <0.05.

## Data Availability

The data that support the findings of this study are available from the corresponding author upon reasonable request.

## References

[1] World Health Organization (2024). Global Tuberculosis Report 2024.

[2] Vasiliu A., Martinez L., Gupta R.K., Hamada Y., Ness T., Kay A., Bonnet M., Sester M., Kaufmann S.H.E., Lange C. (2023). Tuberculosis prevention: Current strategies and future directions. Clin. Microbiol. Infect..

[3] Houben R.M., Dodd P.J. (2016). The Global Burden of Latent Tuberculosis Infection: A Re-estimation Using Mathematical Modelling. PLoS Med..

[4] Cui X., Gao L., Cao B. (2020). Management of latent tuberculosis infection in China: Exploring solutions suitable for high-burden countries. Int. J. Infect. Dis..

[5] Flynn J.L., Chan J. (2001). Tuberculosis: Latency and reactivation. Infect. Immun..

[6] Yang Q., Qi F., Ye T., Li J., Xu G., He X., Deng G., Zhang P., Liao M., Qiao K. (2023). The interaction of macrophages and CD8 T cells in bronchoalveolar lavage fluid is associated with latent tuberculosis infection. Emerg. Microbes Infect..

[7] Silver R.F., Xia M., Storer C.E., Jarvela J.R., Moyer M.C., Blazevic A., Stoeckel D.A., Rakey E.K., Tennant J.M., Goll J.B. (2023). Distinct gene expression signatures comparing latent tuberculosis infection with different routes of Bacillus Calmette-Guerin vaccination. Nat. Commun..

[8] Zhang Y., Zhan B., Hao X., Wang W., Zhang X., Fang C., Wang M. (2023). Factors associated with diagnostic delay of pulmonary tuberculosis among children and adolescents in Quzhou, China: Results from the surveillance data 2011–2021. BMC Infect. Dis..

[9] Chen Q., Yu S., Rui J., Guo Y., Yang S., Abudurusuli G., Yang Z., Liu C., Luo L., Wang M. (2022). Transmissibility of tuberculosis among students and non-students: An occupational-specific mathematical modelling. Infect. Dis. Poverty.

[10] Jenkins H.E., Tolman A.W., Yuen C.M., Parr J.B., Keshavjee S., Perez-Velez C.M., Pagano M., Becerra M.C., Cohen T. (2014). Incidence of multidrug-resistant tuberculosis disease in children: Systematic review and global estimates. Lancet.

[11] Zhang M., Wang T., Hou S., Ye J., Zhou L., Zhang Z., Deng Z., Da Q., Li G., Li S. (2019). An Outbreak of Multidrug-Resistant Tuberculosis in a Secondary School—Hubei Province, 2019. China CDC Wkly..

[12] Yuan Y., Jin J., Bi X., Geng H., Li S., Zhou C. (2023). Factors associated with refusal of preventive therapy after initial willingness to accept treatment among college students with latent tuberculosis infection in Shandong, China. BMC Infect. Dis..

[13] Chen Q., Wang X.M., Qi Y., Liu X.F., Jiang L.P., Hou W., Zhou L., Lu X.W. (2015). The Impact of Directly Observed Therapy on Preventive Treatment for Latent Tuberculosis Infection among Students in Dalian, China. Biomed. Environ. Sci..

[14] Lonnroth K., Migliori G.B., Abubakar I., D’Ambrosio L., de Vries G., Diel R., Douglas P., Falzon D., Gaudreau M.A., Goletti D. (2015). Towards tuberculosis elimination: An action framework for low-incidence countries. Eur. Respir. J..

[15] Getahun H., Matteelli A., Abubakar I., Aziz M.A., Baddeley A., Barreira D., Den Boon S., Borroto Gutierrez S.M., Bruchfeld J., Burhan E. (2015). Management of latent Mycobacterium tuberculosis infection: WHO guidelines for low tuberculosis burden countries. Eur. Respir. J..

[16] Halliday A., Whitworth H., Kottoor S.H., Niazi U., Menzies S., Kunst H., Bremang S., Badhan A., Beverley P., Kon O.M. (2017). Stratification of Latent Mycobacterium tuberculosis Infection by Cellular Immune Profiling. J. Infect. Dis..

[17] Sloot R., Schim van der Loeff M.F., Kouw P.M., Borgdorff M.W. (2014). Risk of tuberculosis after recent exposure. A 10-year follow-up study of contacts in Amsterdam. Am. J. Respir. Crit. Care Med..

[18] Drain P.K., Bajema K.L., Dowdy D., Dheda K., Naidoo K., Schumacher S.G., Ma S., Meermeier E., Lewinsohn D.M., Sherman D.R. (2018). Incipient and Subclinical Tuberculosis: A Clinical Review of Early Stages and Progression of Infection. Clin. Microbiol. Rev..

[19] LoBue P.A., Mermin J.H. (2017). Latent tuberculosis infection: The final frontier of tuberculosis elimination in the USA. Lancet Infect. Dis..

[20] Matteelli A., Rendon A., Tiberi S., Al-Abri S., Voniatis C., Carvalho A.C.C., Centis R., D’Ambrosio L., Visca D., Spanevello A. (2018). Tuberculosis elimination: Where are we now?. Eur. Respir. Rev..

[21] Pollock K.M., Whitworth H.S., Montamat-Sicotte D.J., Grass L., Cooke G.S., Kapembwa M.S., Kon O.M., Sampson R.D., Taylor G.P., Lalvani A. (2013). T-cell immunophenotyping distinguishes active from latent tuberculosis. J. Infect. Dis..

[22] Takenami I., Loureiro C., Machado A., Emodi K., Riley L.W., Arruda S. (2013). Blood Cells and Interferon-Gamma Levels Correlation in Latent Tuberculosis Infection. ISRN Pulmonol..

[23] Potter N.S., Harding C.V. (2001). Neutrophils process exogenous bacteria via an alternate class I MHC processing pathway for presentation of peptides to T lymphocytes. J. Immunol..

[24] Svensson L., Wenneras C. (2005). Human eosinophils selectively recognize and become activated by bacteria belonging to different taxonomic groups. Microbes Infect..

[25] Prakash Babu S., Narasimhan P.B., Babu S. (2019). Eosinophil Polymorphonuclear Leukocytes in TB: What We Know so Far. Front. Immunol..

[26] Bohrer A.C., Castro E., Tocheny C.E., Assmann M., Schwarz B., Bohrnsen E., Makiya M.A., Legrand F., Hilligan K.L., Baker P.J. (2022). Rapid GPR183-mediated recruitment of eosinophils to the lung after Mycobacterium tuberculosis infection. Cell Rep..

[27] Wang Z., Guo Z., Wang W., Zhang Q., Song S., Xue Y., Zhang Z., Wang J. (2025). Prediction of tuberculosis treatment outcomes using biochemical makers with machine learning. BMC Infect. Dis..

[28] Sui X., Xie T., Xu Y., Zhang A., Zhang Y., Gu F., Li L., Xu Z., Chen J. (2023). Protease-Activated Receptor-2 and Phospholipid Metabolism Analysis in Hyperuricemia-Induced Renal Injury. Mediat. Inflamm..

[29] Kwan P.K.W., Periaswamy B., De Sessions P.F., Lin W., Molton J.S., Naftalin C.M., Naim A.N.M., Hibberd M.L., Paton N.I. (2020). A blood RNA transcript signature for TB exposure in household contacts. BMC Infect. Dis..

[30] Hu Y., Zhao Q., Wu L., Wang W., Yuan Z., Xu B. (2013). Prevalence of latent tuberculosis infection and its risk factors in schoolchildren and adolescents in Shanghai, China. Eur. J. Public Health.

